# Virtual reality for the rehabilitation and prevention of intimate partner violence – From brain to behavior: A narrative review

**DOI:** 10.3389/fpsyg.2022.788608

**Published:** 2023-04-27

**Authors:** Tania Johnston, Sofia Seinfeld, Cristina Gonzalez-Liencres, Nicolas Barnes, Mel Slater, Maria V. Sanchez-Vives

**Affiliations:** ^1^Institut d’Investigacions Biomèdiques August Pi i Sunyer (IDIBAPS), Barcelona, Spain; ^2^Event Lab, Department of Clinical Psychology and Psychobiology, University of Barcelona, Barcelona, Spain; ^3^Image Processing and Multimedia Technology Center, Universitat Politècnica de Catalunya-Barcelona Tech, Terrassa, Spain; ^4^Max Planck School of Cognition, Leipzig, Germany; ^5^Departament de Justícia, Generalitat de Catalunya, Barcelona, Spain; ^6^Institute of Neurosciences, University of Barcelona, Barcelona, Spain; ^7^ICREA, Barcelona, Spain

**Keywords:** intimate partner violence, virtual reality, prevention, rehabilitation, perpetrators, empathy, attitudes, embodiment

## Abstract

Rehabilitation and prevention strategies to reduce intimate partner violence (IPV) have limited effectiveness in terms of improving key risk factors and reducing occurrence. Accumulated experimental evidence demonstrates that virtual embodiment, which results in the illusion of owning a virtual body, has a large impact on people’s emotional, cognitive, and behavioral responses. This narrative review discusses work that has investigated how embodied perspective - taking in virtual reality has been used as a tool to reduce bias, to enhance recognition of the emotional state of another, and to reduce violent behaviors, in particular in the realm of IPV. Some of the potential neurological mechanisms behind these affective and behavioral changes are also discussed. The process of rehabilitation and prevention is complex and not always effective, but the integration of neuroscience-inspired and validated state-of-the-art technology into the rehabilitation process can make a positive contribution.

## Introduction

Intimate partner violence (IPV), is defined by the World Health Organization (WHO) as any “behavior within an intimate relationship that causes physical, sexual or psychological harm, including acts of physical aggression, sexual coercion, psychological abuse and controlling behaviors” (Mikton, 2010). It constitutes a global public health issue (World Health Organization, 2013; European Union Agency for Fundamental Rights, 2014) and has an adverse impact on survivors’ physical and psychosocial health (Campbell, 2002; Ellsberg et al., 2008) as well as on society and victims (National Center for Injury Prevention and Control, 2003). For this reason, its prevention is considered of paramount importance and is a priority.

Current prevention strategies include primary and secondary approaches, which are concerned with modifying attitudes and behaviors of the public, before violence has occurred (hereafter referred to as prevention) and tertiary prevention, which involves working with perpetrators (hereafter referred to as rehabilitation).

Prevention strategies usually focus on raising awareness, modifying attitudes related to violence and to survivors of such violence, and teaching new strategies to avoid or react to violent incidents. A certain number of large-scale prevention programs for young people have been developed, evaluated, and implemented in schools and universities, some of which had a positive impact on victimization and re-victimization (see for instance Foshee et al., 2005; Wolfe et al., 2009; Taylor et al., 2015). Rehabilitation strategies usually focus on modifying attitudes towards gender norms and towards women, but also on specific criminogenic needs of perpetrators such as emotion management and empathy among others (see for instance Babcock et al., 2004; Feder and Wilson, 2005). Although overall, rehabilitation strategies have been found to be effective to some extent (Jolliffe and Farrington, 2007), their efficacy remains equivocal with some interventions having a rather small effect or no significant impact on treatment outcomes (Stover et al., 2009; Eckhardt et al., 2013).

In sum, both prevention and rehabilitation approaches still need to improve their effectiveness (Ellsberg et al., 2015). In this context, virtual reality (VR) might be a potentially interesting therapeutic tool for the prevention and rehabilitation of violence - related behaviors, in fields like gender violence, and—in the topic of the present review—IPV. VR allows implicit and experiential learning and people react realistically to VR scenarios and interactions (see Sanchez-Vives and Slater, 2005; Slater, 2009; Slater and Sanchez-Vives, 2016 for a review).

The purpose of this narrative review is to provide an overview of how VR is being used for the rehabilitation and prevention of IPV. First, different VR mechanisms used for prevention and rehabilitation of violence will be introduced, namely the illusions of presence and plausibility and the processes of embodiment and perspective taking. Then, the advantage of these mechanisms as well as practical applications of such a methodology in the field of rehabilitation and of prevention of IPV will be explored. Finally, the neural and behavioral mechanisms that are at play will be discussed.

## VR effects relevant for the prevention and rehabilitation of IPV

### Presence and plausibility in VR for the study of violent behaviors

In VR, people can experience a strong perceptual illusion of presence and plausibility (Slater, 2009). Presence refers to the feeling of “being there,” therefore feeling that one is really inside the scene represented by the VR, while plausibility refers to the subjective perception that the events and situations taking place in the VR scene are really happening. Evidence shows that presence occurs as a result of VR supporting the same sensorimotor contingencies for perception as those we use in the real world to perceive. Sensorimotor contingencies refer to non-conscious automatic rules that we follow for perception—for example, to see underneath something we bend down and move our head and eyes in the appropriate positions and orientations (O’Regan and Noë, 2001; Noë, 2004). If we use our body to perceive in VR much the same as we perceive in reality, then the most logical hypothesis for the brain to adopt is that what we perceive is really happening.

Plausibility is thought to be mainly influenced by virtual events responding to participants’ actions and also on the occurrence of contingent events that relate to the participants (e.g., a virtual human character spontaneously speaking to the participant).

The fact that in VR, participants tend to respond realistically, has been leveraged to study situations that, for practical or ethical reasons, would not be possible in reality. This offers a particular advantage, especially in the study of violence and its correlates. For instance, the Obedience to Authority experiments carried out by Milgram (1974) cannot be repeated today because of ethical concerns. However, VR has been used to virtually reproduce these studies, finding similar results to the original studies (Slater et al., 2006), and has also been used to study how obedience is impacted by identification with the victim (Gonzalez-Franco et al., 2018). Similarly, the bystander problem—whether people intervene to help the victim of a violent situation—cannot be experimentally studied in reality, as it would require violence between people, but it has been studied in VR (Slater et al., 2013; Rovira et al., 2021; Johnston 2021) and the current literature on this topic has been reviewed in Xue et al. (2021).

In the following section, we will explain in detail why in VR it is possible to experience a strong illusion of owning a virtual body (i.e., embodiment), even when having a drastically different visual appearance compared with the subject’s own real body, and how this change of bodily-self and perspective-taking simulations might lead to attitudinal, cognitive, and behavioral changes.

### Embodiment and perspective-taking in VR

The internal representation of the body can be modified depending on the sensory afferents from the body, or sensorimotor correlations during body actions, and this can generate body illusions. Body illusions may include the sensation of a transformed body (e.g., alteration in size or shape), or of movement or an altered position. A variety of such body illusions had earlier been described by, for example Lackner (1988), although the rubber hand illusion of Botvinick and Cohen (1998) is the most well-known. Researchers in the field of VR started reproducing these illusions in the early 2000s using a virtual body in immersive VR and visuo-tactile correlations. In a first implementation carried out by Slater et al. (2008), participants saw a virtual arm emerging from their shoulder in full stereo 3D with their corresponding real hand out of sight. Their real hand was stimulated by an experimenter tapping it with a tracked controller and this was translated into a virtual ball tapping the person’s virtual hand. An asynchronous condition was also included. The results obtained were very similar to those of the original Botvinick and Cohen (1998) rubber hand illusion study in terms of experiencing the subjective illusion of ownership of the arm and the occurrence of a proprioceptive drift towards the virtual arm. Sanchez-Vives et al. (2010) reproduced the virtual hand illusion for correlated real and virtual hand movements, making use of visuomotor and proprioceptive correlations. This approach was eventually extended to the full body ownership illusion by Slater et al. (2010). It was found that the strongest modifier of the subjective illusion of body ownership was perspective position, with the first-person perspective leading to higher ratings of ownership than the third-person perspective, though there was also contribution from synchronous visuomotor and visuotactile stimulation (Petkova et al., 2011; Maselli and Slater, 2013).

**Figure 1:**
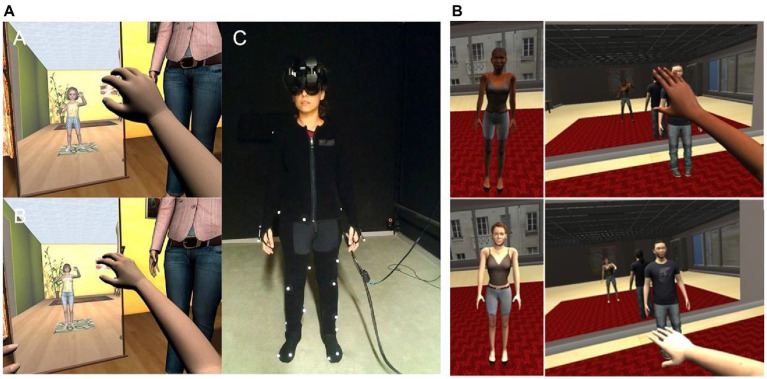
Embodiment in different bodies. **(A)** Screenshots of the child embodiment scene. A. From the first-person perspective of a virtual child. B. From the perspective of a virtual adult body shrunk to the same size as the child. C. Embodiment in a White or Black virtual body instructed to follow the movements of a Tai Chi teacher. **(A)** Source: Banakou et al. (2013). Reproduced with permission. **(B)** Source: Banakou et al. (2016) (CC-BY 4.0).

The form (i.e., visual appearance) of the embodied virtual body, towards which participants are experiencing a body ownership illusion, has been shown to have consequences on physiological responses, behaviors, and implicit attitudes. For example, the size and type of the embodied body can affect how we perceive the world around us. Banakou et al. (2013) showed that when adults are embodied in a virtual body of a child of about 4 years old, they overestimate object sizes by around double compared with a group that are embodied in an adult-shaped body with the same height of the child, demonstrating a cognitive impact of body shape in addition to that of body size (Figure 1A), an effect that has also been replicated in Tajadura-Jiménez et al. (2017).

Furthermore, the body that we inhabit can influence our attitudes and behaviors. For example, people who embodied a taller body than that of their real height became more confident and aggressive in a negotiations task compared with a control group (Yee et al., 2009). The authors referred to this as the Proteus effect (Yee and Bailenson, 2007; see Ratan et al. (2020) for a recent meta-analysis). This has many implications, such as the ones found in the study of Kilteni et al. (2013), where being embodied either in a light-skinned formally dressed virtual character or in a dark-skinned casually dressed avatar (i.e., reminiscent of a rock musician), modulated the way people played drums in the real-world, while simultaneously seeing themselves playing virtual drums in an immersive virtual environment. It was found that the amount of body movement was greater for those participants in the casually dressed dark-skinned virtual character compared to those in the light-skinned formally dressed avatar. Moreover, the movement scores positively correlated with the level of subjective body ownership in the casually dressed condition. Here the type of virtual body influenced measurable behavior.

Therefore, altering the perception of the self, starting from a purely bodily aspect, seems to modify self-related socio-cognitive processing which can be potentially exploited in order to tackle real-life problems such as prejudice. Virtual embodiment through immersive VR can be used as a “perspective-taking tool” that allows individuals to be put in the shoes of different virtual characters and see things from their perspective. This methodology has been used to decrease racial prejudice. For instance, Peck et al. (2013) found that using immersive VR to embody light-skinned participants in a dark-skinned body decreased implicit racial bias towards this specific out-group. This was evidenced by a reduction in an implicit measure of racial prejudice [for a review on VR and racial bias, see Maister et al. (2015)]. But can these experiences in VR have an impact that persists after VR? Banakou et al. (2016) embodied participants either in a black or white body while they practiced virtual Tai Chi (Figure 1B). Implicit racial bias was tested 1 week before and 1 week after the virtual embodiment experience. It was found that the reduction in racial implicit bias was maintained 1week after participants’ exposure to the scenario, and that more than one exposure seemed to enhance these effects. The racial bias results have been further replicated by Banakou et al. (2020). Embodiment in virtual avatars has also been used to increase empathy and decrease prejudice against marginalized groups such as people with disabilities (Chowdhury et al., 2021), people with HIV (Toppenberg et al., 2019), or even homeless people (van Loon et al., 2018).

Close to the subject of the current paper, both non-offender male controls and convicted gender violence offenders have experienced in VR the situation of being abused in simulated situations related to IPV, but while experiencing the embodied perspective of a female victim (Seinfeld et al., 2018). Similarly, United States police have experienced the situation of being a victim of a racially aggravated interrogation by police embodied as a black suspect (Kishore et al., 2021), and men have experienced the situation of being sexually harassed from the embodied viewpoint of a woman, both in computer graphics based VR (Neyret et al., 2020) and in 360° VR (Ventura et al., 2021).

In conclusion, the embodiment of a virtual body, its aspect and experiences, can have an influence on the perception of the world and other people, and further, on one’s own attitudes and behaviors, with different brain mechanisms underlying these effects (de Borst et al., 2020; Seinfeld et al., 2021). In addition, it provides us with the capability of experiencing a virtual situation from the embodied perspective of a different character. This can be used as a useful tool to facilitate the understanding and experience of others. For this reason, it is a promising tool in the work for rehabilitation and prevention of IPV.

To date, only a few papers have been published on the specific topic of the use of VR in the field of IPV: five studies were published on the theme of VR and IPV (Seinfeld et al., 2018, 2021, 2023; de Borst et al., 2020; Gonzalez-Liencres et al., 2020) and one published paper gives a perspective on the use of VR in rehabilitation with IPV (Barnes et al., 2022). Extending the theme of bystander behavior in IPV contexts, two more studies have been carried out related to the evaluation of bystander behavior in cases of dating violence (Jouriles et al., 2016a,b).

Opening the search to other topics more globally related to gender violence and VR, including 360° video as a VR media, several themes are beginning to be investigated. These are concerned with assessment of key factors in the perpetration of violence and with the modification of emotions, behaviors or attitudes, through virtual perspective-taking and role playing. More specifically, sexual harassment prevention has been explored through perspective-taking experiences in VR, by giving male participants the visual point of view of the victim in order to improve their attitudes towards the subjects (Steinfeld, 2020; Sadeh-Sharvit et al., 2021; Ventura et al., 2021; Wu et al., 2021). VR has also been applied to the topic of sexual violence, where a study has explored how VR could help sexual violence survivors heal (Lee et al., 2021). Further research has used VR to improve prevention, for instance through the use of embodied realistic role-playing in order to teach young woman rape-resistance skill in simulated situations (Jouriles et al., 2009).

Departing from the specific field of gender violence, but relevant to this review, VR is a tool that has also been used for the evaluation of deviant sexual preferences in sexual offenders (see for instance Renaud et al., 2002; Trottier et al., 2019). VR has more generally been used also in the field of forensic psychiatry (see reviews in Benbouriche et al., 2014; Sygel and Wallinius, 2021).

In summary, we can see that VR is being used for the study of behaviors, emotions, and cognitions related to violence, but because VR also makes it possible to put users in situations of violence that they experience from a different body or perspective, it is also being used to modify such behaviors, emotions, and thoughts.

In the following sections, we will review in more detail research focused on the prevention and rehabilitation of IPV based on the use of embodiment and perspective-taking in realistic simulated situations in VR.

## VR for the rehabilitation of IPV offenders

### Taking the perspective of the female victim in an IPV agression

Many rehabilitation programs for domestic violence include empathy training or perspective-taking techniques with the main goal of helping people with a history of domestic violence perpetration to understand the feelings and negative consequences suffered by the victims (van Berkhout and Malouff, 2016). Such techniques include watching movies related to domestic violence, reading victim testimonies, or engaging in role-playing activities where aggressors can take the perspective of a victim in a simulated situation. The inclusion of these perspective-taking techniques in offender rehabilitation programs is based on theoretical models that posit that offenders have difficulties recognizing negative emotions in their victims, which hinders their compassionate responses and significantly impacts their emotion-regulation capabilities (Jolliffe and Farrington, 2004; Marsh and Blair, 2008; Van Langen et al., 2014). For instance, there is evidence that difficulties in recognizing fear in female faces is positively correlated with the occurrence of IPV (Marshall and Holtzworth-Munroe, 2010).

There are several drawbacks associated with these traditional perspective-taking techniques. One is that they rely on offenders’ motivation to put themselves in the victim’s shoes, despite the fact that in numerous cases enrolment in rehabilitation programs is court mandated rather than voluntary (Tutty et al., 2020). Moreover, the effectiveness of such techniques also depends on the offender’s imagination capabilities, which varies between individuals. Finally, these techniques are mainly based on giving explicit verbal information against violence, rather than in providing experiences where users can actively learn the negative consequences of violence. For this reason, VR represents an ideal tool for overcoming such limitations, since it enables users to actually experience an abuse situation from the first-person perspective of the victim, independently of motivational factors and of their imagination skills.

**Figure 2:**
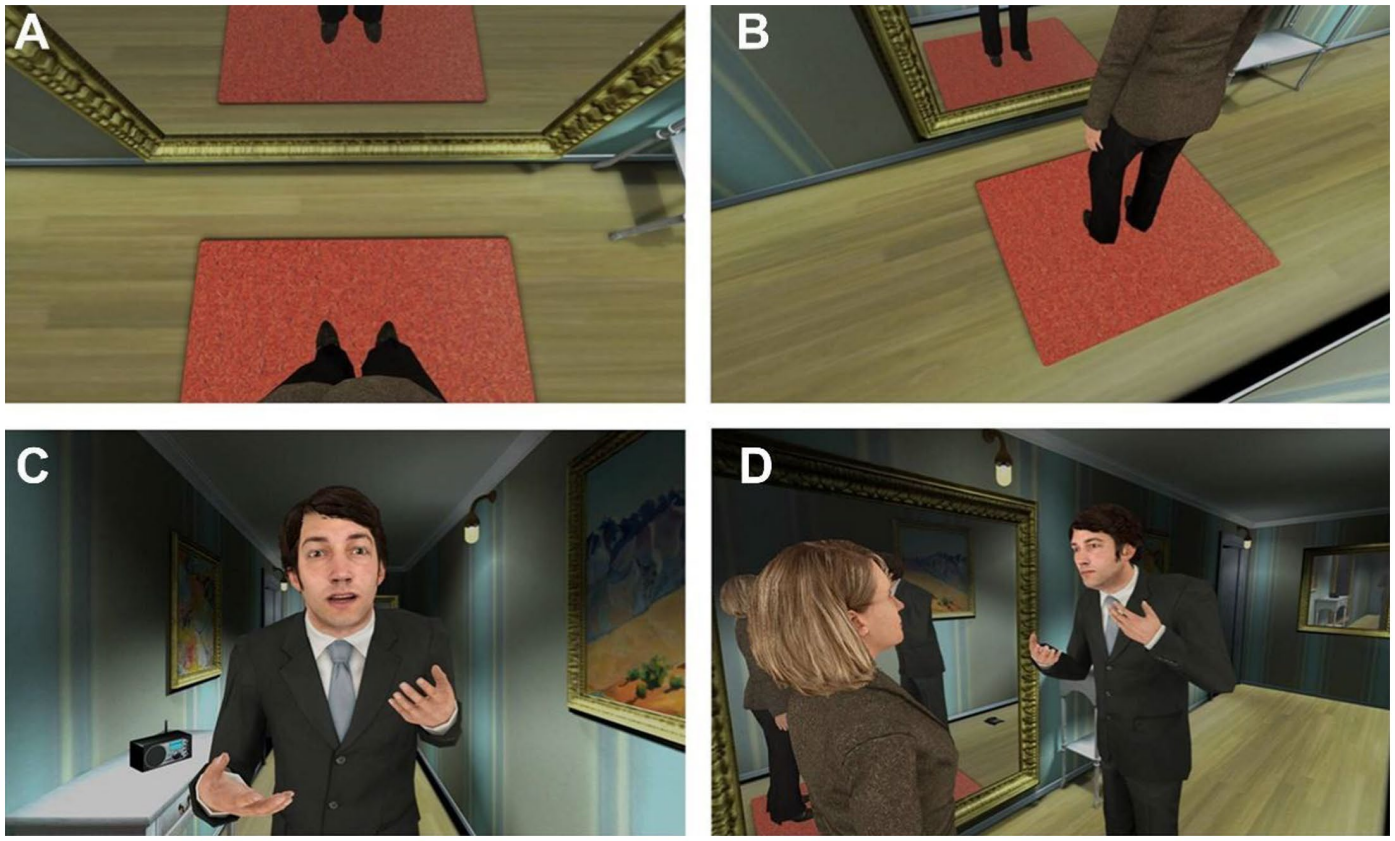
First- versus third-person perspective. **(A)** The participant looks down towards his body and sees the virtual body. (B) A third-person perspective (with no body) over the scenario, where the female character is looking towards a mirror. **(C)** The virtual abuser invades the space of the participant who is embodied as the woman. **(D)** A third person view of the situation shown in **(C)**. Source: Gonzalez-Liencres et al. (2020) (CC-BY 4.0).

**Figure 3:**
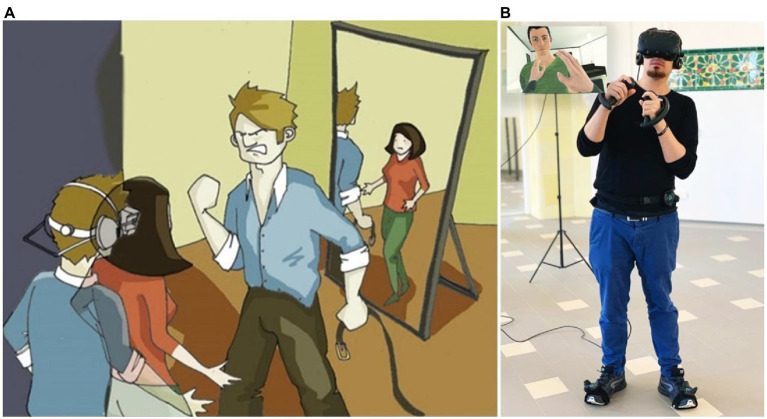
In the body and the perspective of the victim. **(A)** Scheme of the situation in which a male is wearing a head-mounted display to embody a female who is a victim of intimate partner violence. Drawing made by Elias Giannopoulos. **(B)** Image of a participant using a head-mounted display and immersed in the situation depicted in **(A)**. In an inset, what the participant is viewing from his perspective, including his own hands.

Despite the numerous studies that have shown the potential of embodied perspective-taking in VR for changing attitudes and behaviors (Maister et al., 2015), only recently have these methodologies been tested in real clinical settings related to domestic violence. Seinfeld et al. (2018) carried out a study where a group of males with a history of IPV perpetration and a control group of non-offenders experienced a VR scene from the first-person perspective of a female victim of domestic violence (Figure 2). More specifically, in this VR experience when participants looked down towards their own real body, they saw that it was substituted by a life-size female virtual body. Additionally, thanks to the use of full-body tracking devices, participants were also able to control the movement of the virtual body based on their real-time body movements, further enhancing their sense of body ownership and agency over the virtual body (Kilteni et al., 2012). From the female avatar perspective, offenders and the control group of non-offenders experienced a male avatar verbally assaulting them, while progressively getting closer and invading their personal space (Figure 3). At some point in the scene, the male avatar also threw a telephone to the floor towards the victim as an act of physical violence. Before and after the scene, participants completed a computer-based emotion recognition test, where they had to quickly and correctly detect the emotion depicted by faces placed on bodies that expressed emotions that were either congruent or incongruent with the emotional expression depicted by the faces. It was found that at baseline (i.e., before VR), domestic violence offenders had a deficit in recognizing fearful female faces compared with controls, and that this deficit significantly improved after being embodied in the perspective of a female victim of domestic violence in VR (Seinfeld et al., 2018). Moreover, it was also found that the VR experience reduced offenders’ response bias towards wrongly attributing happy emotional states to fearful facial expressions of male and female stimuli. These results highlight the potential of performing embodied perspective-taking exercises in VR to enhance empathy and emotion recognition in rehabilitation programs for IPV perpetrators.

However, the mechanisms underlying the socio-cognitive changes observed after being embodied in a victim perspective in VR remain unclear. Some of these mechanisms were elucidated in a follow-up study, in which the same VR setup was used in order to investigate the behavioral and physiological reactions and subjective feedback of three groups of men: convicted intimate partner batterers, non-convicted (volunteer) intimate partner batterers, and a control group without a history of violence (Gonzalez-Liencres et al., 2021). Some differences and some similarities across the three groups were found: first, convicted batterers saw the potential of this VR rehabilitation tool for *other* people (not themselves), but non-convicted batterers considered it helpful for themselves; second, convicted batterers expressed feelings reminiscent of those of an observer of the abuse scene (e.g., anger, uncertainty, fear), while non-convicted batterers and controls described feelings exemplifying those of the victim (e.g., humiliation, inferiority, desire to cry, intimidation, fear); and third, convicted, and to a lesser degree non-convicted, batterers tended to react behaviorally to more threatening stimuli and their reactions tended to be more emotional than those of controls, even though a larger proportion of controls reacted in general (but less emotionally) to the stimuli in the virtual experience. The results from this study underscored the need to take into account the perpetrators’ profile before assigning them to a particular rehabilitation program, and brought insights into the mechanisms through which changes may occur after embodying a victim in VR.

### Taking the perspective of a child witnessing an IPV situation between their parents

**Figure 4:**
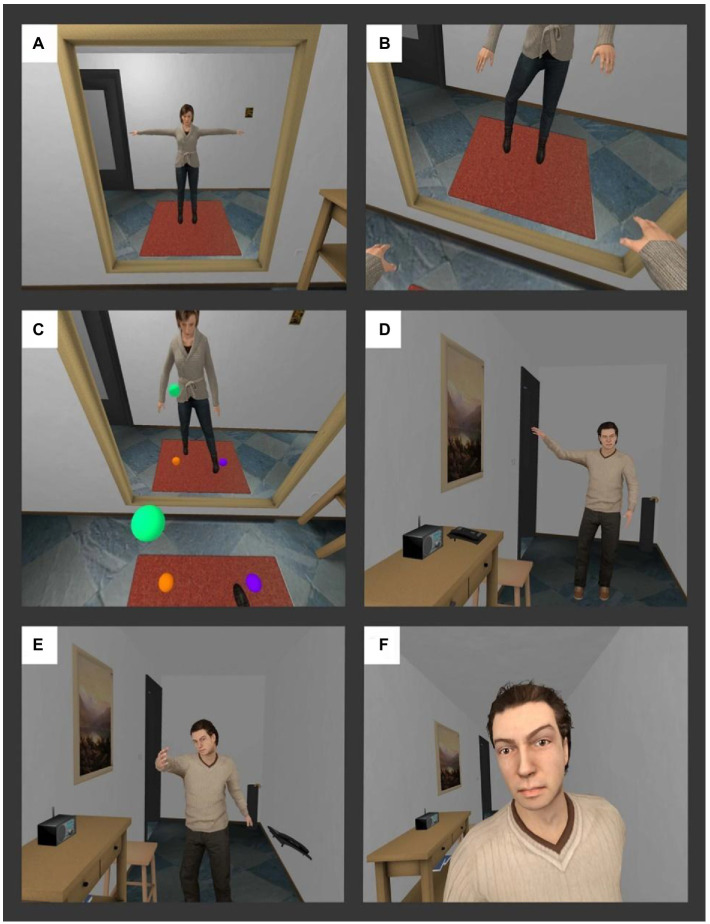
Virtual contents of a scene of intimate partner violence used in Seinfeld et al. (2018). **(A)** The male participant is embodied as a woman by moving his body and having correct sensorimotor correlations, while seeing the virtual body in the mirror. **(B)** The participant’s movements are applied to the virtual body. **(C)** The participant has a task to touch the virtual balls so that they move and see their virtual body movements both directly and in the mirror. **(D)** The male character enters the scene and starts to criticize the participant. **(E)** He pushes a telephone from the counter onto the floor while screaming at the participant. **(F)** He invades the space of the participant. Source: Seinfeld et al. (2018) (CC-BY 4.0).

Abused women frequently report that their children were also exposed to the violent behaviors of their partner, inducing several long-term negative consequences on their physical and psychological health (Holt et al., 2008). Moreover, men who have perpetrated domestic violence usually continue playing an active role in the lives of these children (Salisbury et al., 2009). For this reason, rehabilitation programs for domestic violence include approaches that aim to promote the learning of positive parenting skills, and to help offenders understand the perspective of children (Labarre et al., 2016). There is evidence that domestic violence offenders find it difficult to take the perspective of their children and frequently lack awareness of the negative impact of their aggressive behaviors in children who witness their violent behaviors (Stover and Spink, 2012) (see Figure 4).

**Figure 5:**
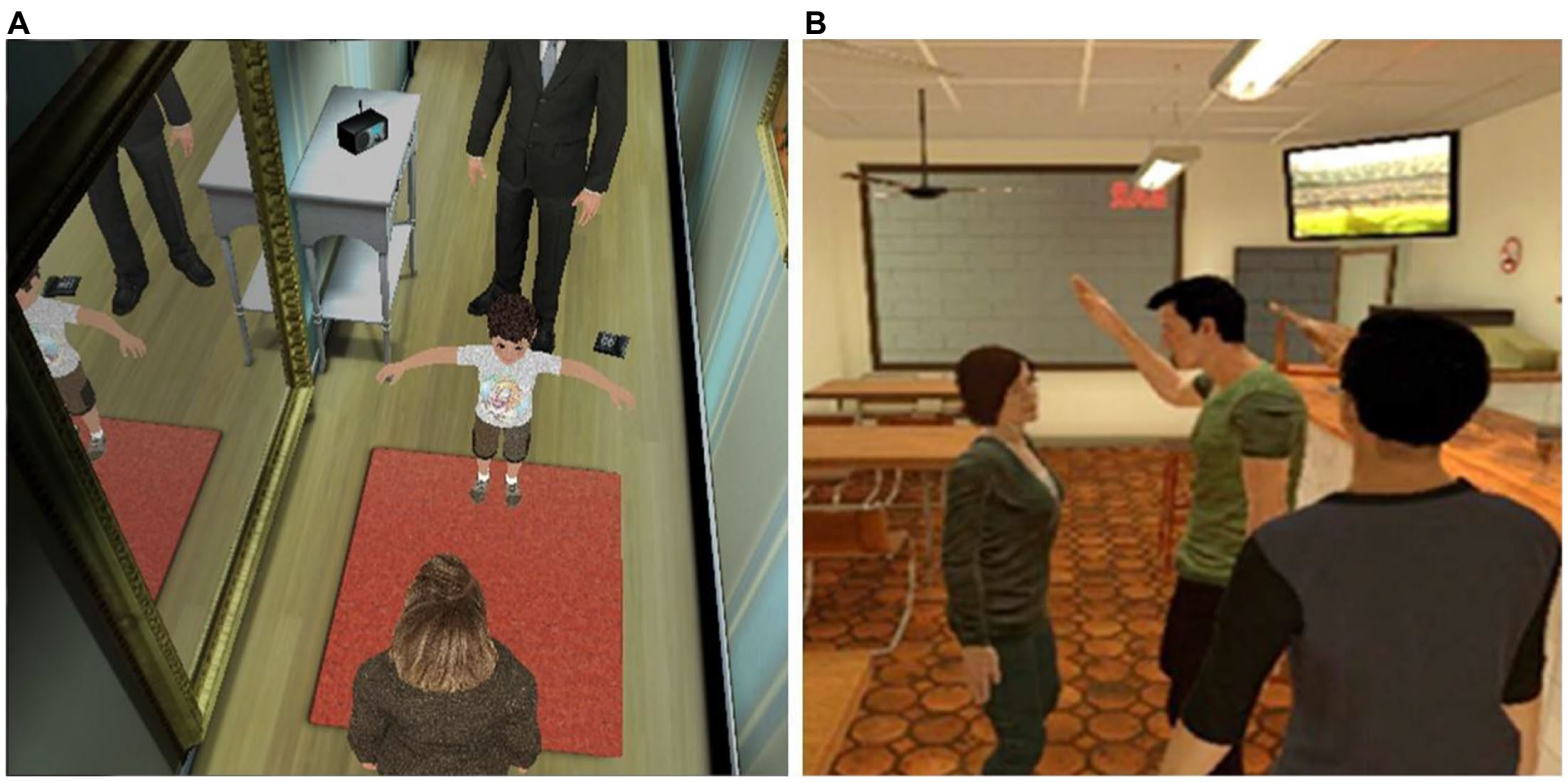
Studies using virtual environments displaying intimate partner violence that is experimented from two different perspectives. **(A)** The participant is embodied as a child and witnesses the abuse by the male avatar towards the female (still taken from the experiment described in Seinfeld et al. (2023)). **(B)** A scenario in a bar where the participant witnesses an attack by a virtual man on his female partner. This is shown from a third-person perspective from behind the virtual body of the participant. Source: Johnston et al. (2021). Reproduced with permission.

As in Seinfeld et al. (2018), where offenders virtually experienced the perspective of a female adult victim of an IPV confrontation, embodied perspective-taking in VR also shows promise for increasing empathy for children and raising awareness of the negative consequences that such behaviors have in this population. For instance, coming back to the study of Banakou et al. (2013), embodiment of adults in a child avatar body not only results in participants overestimating the sizes of objects, but also in self-attributing child-like features, an effect not observed when participants embody an adult avatar shrunk to the same size as the child. In a further study, Hamilton-Giachritsis et al. (2018) evaluated the embodiment of mothers in a child avatar who interacted with a virtual mother who displayed either a positive or negative parenting style. The authors found that being embodied as a child, thereby taking their perspective, while interacting with a negatively parenting mother, resulted in increased emotion recognition and empathic feelings for children. Finally, Seinfeld et al. (2023) tested the use of the VR scene described in Seinfeld et al. (2018), but this time giving offenders the perspective of a virtual child who witnesses the aggressive behaviors of the male avatar towards the female avatar (see Figure 5, which illustrates different sequences of the VR scene of the VR scene). The results of this study show that the VR experience improves emotion recognition (i.e., fear and anger), depending on factors such as past history of violence, relationship status, and educational background. Moreover, the VR scene also positively impacted offenders’ attitudes towards violence and seemed to increase awareness of the detrimental consequences that violence has for children.

### VR in day-to-day use in offender rehabilitation

The two previous sections show that VR facilitates the improvement of some key features related to the rehabilitation of offenders, namely improving empathy through embodiment and perspective taking. In this section, the question of how VR could be incorporated into the day-to-day rehabilitation of IPV offenders in different settings is explored. Both observed settings, namely probation and prisons, are characterized by disparities in perpetrators’ psychological profiles and criminogenic needs (Herrero et al., 2016) and yet in these settings rehabilitation is often delivered in a group format (see Barnes et al. (2022) for a deeper reflection on the topic).

In a recent study, Barnes (2021), using a similar paradigm as Seinfeld et al. (2018), measured whether offenders in prison also improved their empathic skills after embodying the victim of an IPV abuse: 37 men who were serving prison sentences for different types of gender violence crimes experienced the woman embodiment VR scene described above. Empathy was measured before and after the VR intervention with the Interpersonal Reactivity Index (IRI) questionnaire (Davis, 1980), which evaluates four dimensions of empathy: perspective taking (PT), fantasy (FS), empathic concern (EC), and personal distress (PD). Finally, in order to investigate potential differences in criminal profiles in experiences and outcomes of the VR, socio-demographic and criminal and penitentiary data of the participants (i.e., type and number of crimes, time of sentence) were also collected. The overall results of this study showed a trend towards an improvement in empathy: although there were no improvements in perspective taking nor in personal distress, fantasy and empathic concern scores did significantly increase. Turning to differences between perpetrators, less severe violence profiles tended to show better improvement in empathy, especially on the fantasy subscale and to a lesser degree on empathic concern.

This suggests very interesting lines of research that would go in the direction proposed by other authors, in terms of the existence of different profiles of male aggressors. Indeed, some studies describe two profiles of IPV perpetrators: a first type, who perpetrate violence specifically towards their partner, does not have associated problems and does not have a significant criminal record, and a second type, who exercises violence in a more generalized way and has more complex problems such as substance abuse and with a longer criminal record (Loinaz et al., 2010). In the previously cited studies on empathy in VR (Barnes, 2021; Johnston et al., 2021), a similar differentiation is also established, finding a first group of men who exercise violence in a milder form and another second group with a violent behavior of greater severity and intensity. The results presented above suggest that VR may provide better results for the first group.

In that context, VR tools could play a crucial role as they could allow the personalization of the rehabilitation experience through the viewing of tailored VR content adapted to the individual needs of the perpetrator. With that in mind, a study was recently conducted (Johnston et al., 2021), in which one objective was to increase the intensity of the VR experience through the implementation of fake interoceptive feedback that suggested fear. To that end, a group of male IPV perpetrators in prison were immersed in the virtual scene described above, in which they experienced an IPV event from the perspective of the victim. While half of the participants experienced the scene in exactly the same conditions as in the previous studies, the other half additionally received fake interoceptive feedback consistent with fear during the scene: they heard an accelerated heartbeat in their headset and felt a corresponding vibration on their chest, implemented with a vibration device. Measures were taken before and after the VR, and included, among others, perceived interoceptive feelings (i.e., bodily sensations) measured with the Autonomic Perceptions Questionnaire (APQ) questionnaire (Mandler et al., 1958). After the VR experience, participants reported their subjective emotional experience inside VR and their perceptions of the scene (e.g., perceptions of abuse during the scene; feelings of being vulnerable as a woman during the scene, emotions during the scene).

Participants reported higher interoception scores inside VR than before VR and no effect of the fake feedback was found: regardless of whether they had experienced the fake fear feedback or not, participants seemed to have been more stressed during the VR scene of abuse. However, the fake interoceptive feedback did affect participants’ perceptions of the scene. To summarize, offenders in the fake feedback condition had higher perception of abuse and felt more vulnerable than those who experienced the scene without this feedback. An additional analysis showed that these perceptions of the scene were indeed positively correlated to changes in interoception scores. Another important finding was that participants with longer sentences had better emotion recognition skills and more changes in autonomic perceptions inside the VR: overall, results from this study also suggest that different types of perpetrators, in probation versus in prison, and also within prison samples, might not only have different baseline emotion recognition skills, but also different experiences of the same VR scene.

To conclude, personalizing VR scenes to participants’ needs seems needed and feasible. This can be done through fake interoceptive feedback for instance, which seems to allow the modulation of the emotional experience of the VR. However, the role that the criminological profile of the perpetrators has on VR rehabilitation experiences and outcomes, uncovered in the two cited studies, needs to be further explored.

## VR for the prevention of IPV

In the previous sections, the usefulness of VR for rehabilitation has been highlighted, but while embodiment improves empathy in offenders, does it improve other key factors relative to occurrence of violence, for instance attitudes towards women, or behaviors of bystanders when faced with IPV incidents, which are usually targeted in prevention (Mikton, 2010).

While some work has been done in VR in the field of gender violence prevention in general, as described in the Introduction, the literature is still scarce. This section will describe five studies that relate specifically to the prevention of IPV in VR.

### First-versus third-person perspective in VR for modifying attitudes towards women

How real we perceive our experiences in VR is modulated by multiple factors, one of which is the perspective position. A virtual scene experienced from the first-person perspective of a virtual body generates physiological reactions and subjective sensations reminiscent of real life, whereas witnessing the same virtual scene as an observer without a body or from a third-person perspective, does not induce the same physiological reactions nor the same degree of subjective sensations (Slater et al., 2010). The illusion of body ownership in VR is generated when the surrogate body is experienced from a first-person perspective, and it requires that visuomotor or visuotactile synchrony allow for the expected visual and motor/sensory feedback (Petkova and Ehrsson, 2008; Perez-Marcos et al., 2009; Slater et al., 2010; Kokkinara et al., 2016).

A recent study addressed this question of perspective in the context of IPV (Gonzalez-Liencres et al., 2020). As described below, the impact of a VR paradigm in which men convicted of IPV embody a woman verbally abused by a male avatar, has already been studied (Seinfeld et al., 2018), in particular the impact on emotion recognition capabilities. To further investigate the mechanisms through which these effects were observed, a similar paradigm was used in Gonzalez-Liencres et al. (2020) (Figure 2), albeit with men without a history of violence who were assigned to one of two groups: (1) they embodied a woman from a first-person perspective who was verbally abused by a male avatar, or (2) they witnessed that same abuse scene but as a third-person observer. The study assessed the physiological reactions (skin conductance, as a measure of arousal), behavior (they were filmed while in VR and the videos were later analyzed), implicit gender bias (with the Implicit Association Test) and the subjective perceptions of the VR scene (i.e., through a questionnaire and an interview). A correlation between the magnitude of the physiological reactions to threatening stimuli with the following items of the VR questionnaire was found: how vulnerable they felt for being a woman, the sensation that they could be assaulted, how useful the scene could be for abuser rehabilitation, and how different it would have been to experience the scenario on TV. Moreover, the degree to which they identified with the woman correlated with the decrease in prejudice against women. These correlations were found for all participants pooled together, irrespective of the perspective from which they experienced the scene. However, the first-person condition facilitated taking the scene personally, generated sensations of fear, helplessness, and vulnerability, and tended to induce greater behavioral and physiological reactions. The VR questionnaire, which measured participants’ experience of the VR scene, but also their scores on key VR illusions, revealed high scores in both groups, although they were slightly lower in the third-person condition. Since participants were assigned to either the first- or the third-person condition, but not to both, which suggests that their presence in VR was enough to generate plausibility (Slater, 2009). Altogether, Gonzalez-Liencres et al. (2020) concluded that the potential for rehabilitation of offenders originates from presence and from the identification with the victim, which in turn is more easily, but not exclusively, achieved through a first-person perspective.

Building upon these findings, Johnston (2021) explored whether a first-person perspective of the victim could improve implicit forms of victim blaming—a key target in prevention of IPV—measured through a socio-cognitive process named “infrahumanization.” This is the process of attributing to someone more primary emotions, which are shared with animals, than secondary emotions, which are uniquely human (Demoulin et al., 2004), and has been linked to victim blaming in cases of sexual harassment (Lucarini et al., 2020) and of IPV (Baldry et al., 2015). As in Gonzalez-Liencres et al. (2020), participants were exposed to the VR scene depicting IPV abuse and were either embodied as the victim or were given the perspective of an observer across the room (third-person perspective). Self-reported attitudes towards the scene and towards the victim (explicit attitudes), as well as infrahumanization of the victim were measured (implicit attitudes) after VR exposure: preliminary results showed that while explicit attitudes of victim blaming were higher in the group of participants who were embodied as the victim, their implicit attitudes seemed lower. Hence embodiment as the victim could be used as a tool to improve implicit, deeply rooted attitudes (Wilson et al., 2000) towards victims of IPV, although further work should investigate the consequence that embodiment in the victim might have on explicit, self-reported attitudes.

### Bystander position in a gender-violence attack

In addition to the modification of attitudes, another important target of IPV prevention is the reactions of bystanders when they witness, directly or indirectly, incidents of IPV (Fenton and Mott, 2017). Here too, VR has a potential role to play in improving our understanding of bystander behavior in such contexts, in order to improve interventions.

As explained above, when immersed in virtual environments, people tend to respond realistically, whether in terms of physiological responses, attitudes, or behaviors. This makes VR an excellent tool for working towards personal change, but also to evaluate behaviors in an ecologically valid yet safe environment—an aspect that is paramount in the field of violence.

Bystander reactions to violent events are inherently difficult to investigate experimentally, as they are either measured with high ecological validity, through surveillance camera recordings for instance (Liebst et al., 2019), with the disadvantage of not being able to control for any potential correlates of observed behaviors; or through self-reports of one’s behaviors in past events or in imaginary events (e.g., through vignettes). While self-report methods offer an interesting insight into the topic, they are prone to response distortion, due to social desirability (i.e., the tendency to respond in a way one perceives as desirable, rather than in a genuine manner; see Bowling 2005), especially in the delicate field of IPV, and even more so in offender populations.

One way to combine a high level of experimental control with a high level of ecological validity, in order to understand behavioral reactions to violent incidents, is to employ VR to simulate those incidents. This has successfully been used to investigate bystander behavior in the general public when facing a violent event between two (virtual) football fans (Rovira et al., 2009; Slater et al., 2013). In the same scenario, Rovira and Slater (2022) showed that reinforcement learning, applied to the behaviors of the victim, perpetrator and bystanders, can be used to encourage participants towards helping behavior. Closer to the topic of IPV, VR has also been used to measure bystander behaviors of young people in situations of sexual violence (Jouriles et al., 2016b) and of dating violence (Jouriles et al., 2016a). In the two latter studies, social situations were simulated in which participants were in a car, having a conversation with a virtual agent that was controlled in real time by an actor. These virtual situations all depicted a friend reporting situations of potential sexual or dating violence happening right now (e.g., a friend at a party they are about to leave was very drunk and they saw her go upstairs with a young man she did not know well) and the participants’ bystander behavior was measured through their verbal reactions to the situations they heard (e.g., answering they should go back to the party to check on their friend, do nothing, etc.).

In recent work by Johnston et al. (2021), immersive VR was used to compare bystander reactions of offenders and a control group of non-offenders when confronted with a situation of IPV in a bar (Figure 5B). To that end, the reactions of IPV perpetrators and males with no history of violence (control group) were observed, while immersed in a virtual bar where they witnessed an increasingly violent fight between a couple. Participants were embodied in a male avatar body in this study. Subsequently, the helping behaviors of both groups were compared. Further measures were gathered in relation to social desirability levels and attitudes towards acceptability of IPV, in order to investigate the relation between these variables and participant’s helping behaviors.

The results show that offenders helped significantly less and reported significantly higher attitudes of acceptability of IPV than non-offenders. Secondly, social desirability levels did not affect helping behaviors inside VR. This was the case even though social desirability levels were significantly higher in offenders, for whom scores exceeded the threshold of “faking good” suggested by Lambert et al. (2016). Finally, associations between self-reported acceptability of IPV and helping behaviors went in different directions across the groups. For controls, as expected, less accepting attitudes of IPV were associated with more helping behaviors, but in offenders the opposite association was observed: holding more accepting attitudes towards IPV was associated with more intervention.

Overall, these findings support the usability of VR scenarios for the behavioral study of violence and suggest—at least in non-offending populations—that exposing people to a virtual event of violence could serve as an indirect, behavioral measure of their attitudes towards violence. On the other hand, this discrepancy between offenders’ attitudes towards IPV and their behaviors in such situations, associated with higher levels of social desirability scores confirms the necessity of implicit, behavioral measures. It also highlights the necessity to take into account the criminogenic profiles of the offenders when planning and implementing VR tools for evaluation and rehabilitation.

## Brain mechanisms related to the impact of embodied VR on IPV

The research described in this review shows that VR experiences can modify attitudes and behaviors related to domestic and gender-based violence, as well as influence empathy and emotional recognition abilities. This has been shown through behavioral studies such as those carried out by Seinfeld et al. (2018), Gonzalez-Liencres et al. (2020) and Johnston et al. (2021), where being in the first-person perspective of a virtual victim impacted participants’ emotion recognition skills, attitudes towards violence, and physiological responses. However, the underlying brain mechanisms of these cognitive and behavioral effects are still largely unknown and only recently have studies started to investigate this topic using neuroimaging techniques (Maister et al., 2015).

De Borst and colleagues performed an fMRI study where they demonstrated that being in the victim’s shoes (i.e., from a first-person perspective), as opposed to being an observer of the virtual IPV scene described earlier (i.e., from a third-person perspective), modulates network encoding of the bodily self and peripersonal space. Specifically, in this study it was found that several regions of the fronto-parietal network directly related to body ownership and space representation (e.g., pre-motor cortex, intra-parietal sulcus, supramarginal gyrus, superior parietal lobe, and the primary somatosensory cortex), were more synchronized across participants in the first-person perspective compared with the third-person perspective. Moreover, in this study it was also found that brain activity is more synchronized in the amygdala, a critical region for the processing of threat and fear, when the abuse VR scene is experienced from the first-person perspective with respect to the third-person perspective (de Borst et al., 2020).

In an additional fMRI study, Seinfeld et al. (2021) investigated the neural processing of fear-related stimuli associated with being in the first-person perspective of a female victim of domestic violence in VR. The authors used the same VR scene as in Seinfeld et al. (2018), but this time participants underwent an fMRI scanning session before and immediately after being embodied as the victim. During the fMRI scanning sessions that occurred between the VR experiences, participants passively viewed emotional morphs of faces and bodies changing from an extremely happy expression to a fearful one. The different morph steps, genders and identities included in the fMRI stimuli, were presented in random order. The findings of this study indicate that the virtual abuse experience led to an enhancement of the Default Mode Network (DMN) activity, specifically when processing ambiguous emotion stimuli (i.e., stimuli that do not clearly express happiness or fear, but lie in the middle of the morph continua). However, DMN activity decreased after VR when processing clearly fearful expressions. Moreover, after the virtual experience, it was found that patterns for processing stimuli within and between genders (i.e., male and female faces) became more variable in several DMN regions. Taken together, these results suggest that being in the perspective of a victim in VR might enhance the recognition of emotional ambiguous stimuli and empathy through changes in the brain activity of areas that are directly linked to social cognition processes, autobiographical memories, and perspective-taking, such as the DMN. However, no evidence was found that these emotion recognition changes were mediated by changes in how the visual features of the stimuli were perceived, since no changes in face-encoding areas were found (e.g., fusiform cortex) after going through the VR abuse experience. These findings highlight the impact that embodied perspective-taking in VR has on brain areas related to socio-cognitive processing and to the perception of emotional expressions.

The evidence summarized in these sections shows that embodied VR experiences significantly influence brain regions related to the bodily self and impact brain regions that are critical for the processing of social information, such as the DMN, amygdala, fronto-parietal networks, and the fusiform area. However, there are still few studies in this field and more research is needed to test the replicability and generalizability of these results to other scenes and contexts related to IPV. These brain studies highlight the potential of VR not only for rehabilitation purposes, but also to understand the neural underpinnings of perspective-taking and aggressive behaviors.

## Discussion

This narrative review examined the current state of the use of immersive VR in the context of the rehabilitation of IPV. The key elements reviewed here are embodiment in virtual bodies and embodied perspective-taking. This is a powerful alternative to traditional psychological techniques, which rely on the abilities and motivation of the users to imagine certain situations or the perspective of someone else.

A matter of discussion could be whether embodiment is necessary, since being in a virtual situation of IPV, for example, can deliver a highly immersive experience with other approaches. In this sense, one can compare different media, from 2D movies to 360° videos, and then from interactive and immersive VR to interactive and immersive VR lived through an embodied perspective of the different agents involved. Even when the explicit information delivered can be the same in all of these (e.g., same script and depiction), what we look for with embodiment is an implicit learning that derives from the first-person embodied experience (Banakou et al., 2020). Additionally, there will be an explicit learning that can be conceptualized and explained (e.g., “I have realized how my wife must feel when I talk to her like that”) (Gonzalez-Liencres et al., 2021).

The results that have been described are promising, but it is a young field where intense research is still needed. First, a better understanding of the basis of violent behavior and the underlying physiological mechanisms is needed (Lischinsky and Lin, 2020). Within the realm of rehabilitation, current studies are exploring how VR tools can be systematically integrated into existing traditional rehabilitation programs, how this improves outcomes in terms of key factors, such as empathy and attitudes towards violence, but also in terms of recidivism. Further, how these VR tools can be integrated in primary prevention efforts, with non-offending populations, including teenagers and adults, is also under investigation. The VRperGenere project (www.vrpergenere.com), funded by the European Commission, is currently exploring some of these possibilities, both in the rehabilitation and prevention fronts. Immersive VR has great potential to complement current approaches towards rehabilitation of IPV perpetrators and towards primary prevention and, more generally speaking, can contribute to the fight against the global problem that is IPV, and more broadly, violent behavior.

## Ethics statement

Written informed consent was obtained from the individual(s) for the publication of any identifiable images or data included in this article.

## Author contributions

All authors listed have made a substantial, direct, and intellectual contribution to the work and approved it for publication.

## Funding

This research has been funded by the European Union’s Rights, Equality and Citizenship Programme (2014–2020) under Grant Agreement: 881712 (VRperGenere). MS is supported by the ERC Advanced Grant MoTIVE (#742989). Supported by the Departament de Recerca i Universitats de la Generalitat de Catalunya with the code AGAUR 2021 SGR 01165, Grup NEUROVIRTUAL.

## Conflict of interest

MS-V and MS are founders of Virtual Bodyworks Inc. (now Kiin). The authors receive no payment or financial support by this or any other company. Virtual Bodyworks was not involved in the study design, collection, analysis, interpretation of data, the writing of this article or the decision to submit it for publication.

The remaining authors declare that the research was conducted in the absence of any commercial or financial relationships that could be construed as a potential conflict of interest.

## Publisher’s note

All claims expressed in this article are solely those of the authors and do not necessarily represent those of their affiliated organizations, or those of the publisher, the editors and the reviewers. Any product that may be evaluated in this article, or claim that may be made by its manufacturer, is not guaranteed or endorsed by the publisher.
